# Cell cycle activity of neural precursors in the diseased mammalian brain

**DOI:** 10.3389/fnins.2014.00039

**Published:** 2014-02-27

**Authors:** Sara Bragado Alonso, Max Schulze-Steikow, Federico Calegari

**Affiliations:** DFG–Research Center and Cluster of Excellence for Regenerative TherapiesDresden, Germany

**Keywords:** cell cycle, neural stem cells, oligodendrocyte precursor cell (OPC), neurodegenerative diseases, adult neurogenesis

## Abstract

Basic research during embryonic development has led to the identification of general principles governing cell cycle progression, proliferation and differentiation of mammalian neural stem cells (NSC). These findings were recently translated to the adult brain in an attempt to identify the overall principles governing stemness in the two contexts and allowing us to manipulate the expansion of NSC for regenerative therapies. However, and despite a huge literature on embryonic neural precursors, very little is known about cell cycle parameters of adult neural, or any other somatic, stem cell. In this review, we briefly discuss the long journey of NSC research from embryonic development to adult homeostasis, aging and therapy with a specific focus on their quiescence and cell cycle length in physiological conditions and neurological disorders. Particular attention is given to a new important player in the field, oligodendrocyte progenitors, while discussing the limitation hampering further development in this challenging area.

## Introduction

The study of the cell cycle is one of the most prolific areas in developmental neuroscience with hundreds of publications spanning half a century and contributing new methodologies, basic knowledge and a deeper understanding of brain development and evolution (Fujita, 1962; Schultze and Korr, 1981; Takahashi et al., 1995; Dehay and Kennedy, 2007; Salomoni and Calegari, 2010; Borrell and Calegari, in press).

Cell cycle regulation in itself is a huge field and several reviews already discussed its molecular control during brain development and adulthood (Dehay and Kennedy, 2007; Salomoni and Calegari, 2010; Beukelaers et al., 2011b). As one factor fuelling interest in this area, short cell cycles were found to correlate with a higher proliferative potential of neural precursors at the cellular and tissue level and across phylogeny (Borrell and Calegari, in press). This correlation led to functional manipulations showing that the proliferative potential of neural precursors is increased by shortening their cell cycle while, conversely, lengthening it leads to differentiation and neurogenesis (Calegari and Huttner, 2003; Lange et al., 2009; Pilaz et al., 2009; Artegiani et al., 2011; Beukelaers et al., 2011a).

Considering that the first calculation of the cell cycle during development coincided with the first report on adult neurogenesis five decades ago (Altman, 1962; Fujita, 1962) and that immense efforts are currently invested worldwide in stem cell research and regenerative medicine, it comes as a surprise that cell cycle studies during adulthood, contrary to development, are extremely limited with only a handful addressing the diseased brain. Here we summarize our knowledge on cell cycle parameters of adult neural precursors in physiological and pathological conditions with particular attention to a new player in biomedicine, oligodendrocyte progenitors. This is important to identify potential correlations of biological significance and to identify our gaps in knowledge that the field should address in the years to come.

## Neurogenic precursors

### Cell cycle in physiological conditions

Mammalian NSC generate neurons and glia throughout life within two restricted areas: the subgranular zone (SGZ) of the dentate gyrus and the subventricular zone (SVZ) of the lateral ventricles (Zhao et al., 2008; Kriegstein and Alvarez-Buylla, 2009). In both niches a pool of NSC, progenitors and neuroblasts coexist in a dynamic system in which the production of neurons is regulated by intrinsic and extrinsic factors (Lois and Alvarez-Buylla, 1994; Cameron and McKay, 2001). Similarly to their embryonic precursors (Merkle et al., 2004; Li et al., 2013), adult NSC maintain a radial morphology (Doetsch et al., 1999; Seri et al., 2001), contact blood vessels (Palmer et al., 2000; Tavazoie et al., 2008) and share common markers (Kriegstein and Alvarez-Buylla, 2009). However, in contrast to embryonic development no unique marker has been identified that exclusively labels one, but not others, precursor types (Ming and Song, 2011). In addition, no positive marker of quiescent cells is available to date. These limitations, together with the fact that a significant proportion of NSC are quiescent, makes it remarkably difficult to assess cell cycle parameters during adulthood.

In the SGZ, dividing NSC (type 1) give rise to intermediate progenitors (type 2) that in turn generate neuroblasts (type 3) producing granule neurons (Seri et al., 2001; Kempermann et al., 2004). The significance of adult hippocampal neurogenesis is not fully understood but evidence points to a role in learning and memory (Kempermann, 2008; Deng et al., 2010). With regard to the lineage of hippocampal NSC, studies have calculated that type 1 cells undergo 3–4 asymmetric divisions before becoming postmitotic astrocytes (Encinas et al., 2011) while others have concluded that at least some type 1 cells can self-renew unlimited times throughout life (Bonaguidi et al., 2011). Despite this controversy, studies attempting to measure the cell cycle in the adult hippocampus found that cycling NSC divide every about 1 day (Lugert et al., 2010; Encinas et al., 2011; Brandt et al., 2012) with S being the most variable phase among progenitors (Brandt et al., 2012). In particular, type 1 cells complete the cell cycle in 23 h with an S-phase of 10 h (Brandt et al., 2012). Subsequently, type 2 cells lengthen to 27 h while type 3 shorten again to 23 h. Although G1, G2, and M were not individually measured, cell cycle differences were found to be almost exclusively due to S-phase (Table 1) (Brandt et al., 2012).

**Table 1:**
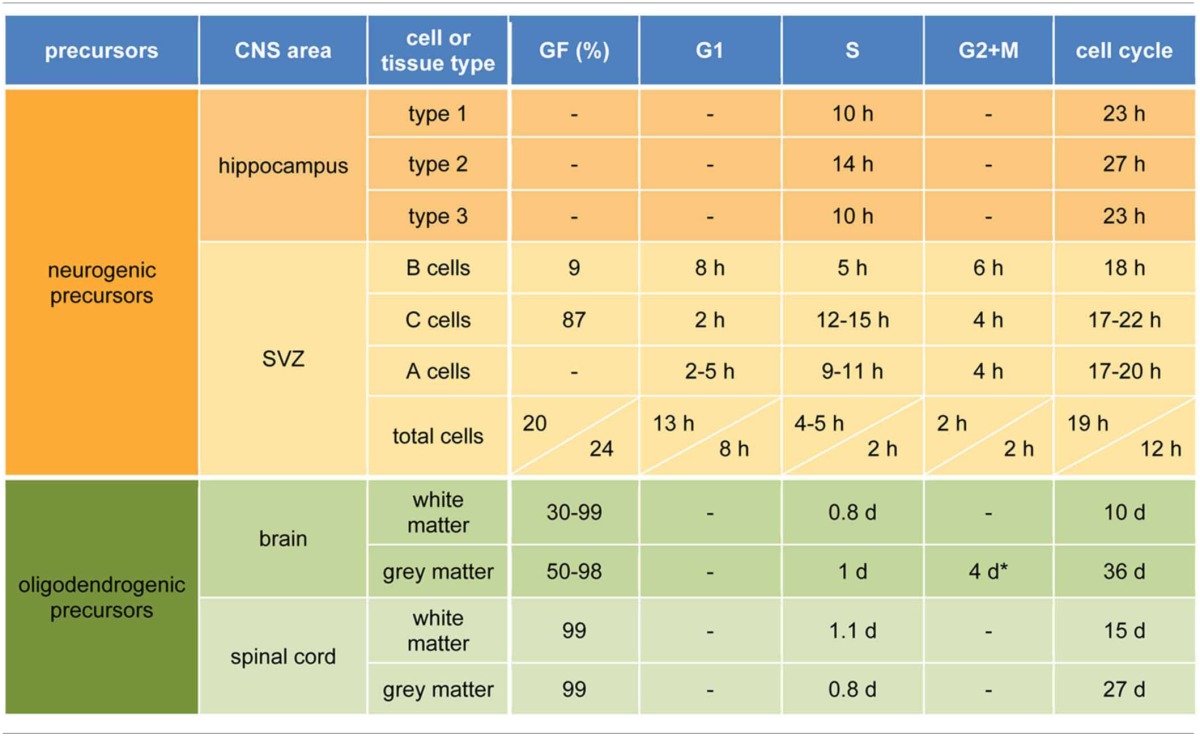
**Cell cycle parameters of neurogenic and oligodendrogenic precursors**.

Left to right: precursor types in different areas of the CNS and individual cell types or tissues are indicated with proportion of cycling cells (growth fraction, GF), length of individual phases and total cell cycle in hours (h) or days (d). Split cells indicate values calculated in physiological (left) or pathological (right) conditions. OPC values were acquired at the age of approximately 2 months. ^*^Value calculated from (Simon et al., 2011; Young et al., 2013).

The SVZ is the second and most proliferative neurogenic niche of the adult mammalian brain (Kriegstein and Alvarez-Buylla, 2009; Ming and Song, 2011). Here, NSC (B cells) have an apical process intercalating between ependymal cells and contacting the ventricle (Mirzadeh et al., 2008) and a basal process contacting blood vessels (Shen et al., 2008). B cells give rise to amplifying progenitors (C cells) that generate migrating neuroblasts (A cells) dividing along the SVZ and rostral migratory stream toward the olfactory bulb where they ultimately differentiate into neurons (Petreanu and Alvarez-Buylla, 2002; Ming and Song, 2011). At any given time, B cells represent 10% of all cycling cells (Doetsch et al., 2002) with a similar proportion being cycling as opposed to quiescent (Ponti et al., 2013). B cells complete the cell cycle in 18 h and G1 and S in 8 and 5 h, respectively (Table 1) (Ponti et al., 2013). Regarding C cells, these represent over 60% of proliferating cells (Doetsch et al., 2002) with nearly 90% of them cycling at any given time (Ponti et al., 2013). C cells are proposed to divide symmetrically 2–3 times before generating A cells and have more heterogeneous cell cycles of 17–22 h, a longer S-phase of 12–15 h and a remarkably short G1 of 2 h (Ponti et al., 2013). Finally, A cells account for 26% of dividing cells in the SVZ (Doetsch et al., 2002), have a cell cycle similar to C cells with perhaps a longer G1 (2–5 h) and shorter S of 10 h (Table 1) (Ponti et al., 2013).

Altogether, cell cycle differences within neurogenic niches seem minor with the only consistent change being a lengthening of S-phase from NSC to progenitors and shortening from progenitors to neuroblasts. Not only is the significance of such changes unknown but no parallelism is evident between embryonic and adulthood precursors because in the former S-phase was found to be longer in NSC than in progenitors (Arai et al., 2011) and G1 during adulthood was found to length from C to A cells but not from B to C cells (Ponti et al., 2013).

### Cell cycle in pathological conditions

Neural progenitors increase their proliferation, meant both as exiting quiescence and shortening the cell cycle, under pathological conditions in both neurogenic niches (Dash et al., 2001; Arvidsson et al., 2002). Most studies focused on the SVZ where neural precursors change their migration and are redirected to the injured area to acquire the phenotype of local cells (Arvidsson et al., 2002), thus, making the SVZ a potential target of therapy. Increased proliferation and altered migration were found in rodent models of multiple sclerosis (Rasmussen et al., 2011; Mecha et al., 2013), Huntington's disease (Tattersfield et al., 2004), Parkinson (Aponso et al., 2008) and stroke (Thored et al., 2006), the latter of which was also shown in humans (Jin et al., 2006; Minger et al., 2007). Among these diseases, the neurogenic response triggered by stroke is the most prominent and best characterized.

Stroke is a cerebrovascular accident resulting in a permanent damage and second leading cause of death worldwide (WHO, 2013). Two days after striatal stroke, SVZ precursors shorten the cell cycle form 19 to 12 h due to a shorter G1 from 13 to 8 h and S from 5 to 2 h (Table 1) (Zhang et al., 2006, 2008). Subsequently, cell cycle progressively lengthens reaching normal values 14 days after stroke. Interestingly, the proportion of proliferative, as opposed to differentiative, divisions increases (from 10 to 50%) during the period of short cell cycles and, conversely, decreases (back to 10%) during the long ones (Zhang et al., 2008) although it is important to note that in these studies precursor types were not distinguished and that these values refer to the total SVZ population. Nevertheless, stroke results in an increasing cohort of neuroblasts migrating from the SVZ toward the striatum with a peak at day 14 (Zhang et al., 2004) and lasting for at least 4 months (Arvidsson et al., 2002; Thored et al., 2006; Yamashita et al., 2006). Most of these newborn neurons undergo apoptosis but those that survive functionally integrate (Yamashita et al., 2006; Hou et al., 2008) with evidence indicating that this endogenous neurogenesis can contribute to functional recovery after stroke since, for instance, ablation of neural precursors impairs recovery (Jin et al., 2010; Sun et al., 2013). Yet, it still has to be shown whether an artificial increase in endogenous neurogenesis would favor brain function.

Altogether, cell cycle parameters of precursor cells after stroke recapitulate embryonic development in the sense that short cell cycles are coupled to proliferation and long cell cycles to differentiation. In this context, cell cycle re-entry is likely instrumental to guide the stroke-induced neurogenic response and, in fact, activation of cell cycle regulators is known to occur in both rodents and humans (Love, 2003; Rashidian et al., 2007). Yet, this response in postmitotic neurons is likely to induce apoptosis rather than cell cycle re-entry (Rashidian et al., 2007). This is supported by the fact that injecting a cdk inhibitor in the ischemic area reduces apoptosis and extension of the ischemic core (Osuga et al., 2000) suggesting that cell cycle regulators have different effects in postmitotic neurons or precursors, which should be considered in manipulations aimed to improve recovery. In this context, increasing the endogenous pool of neurogenic precursors by manipulating their cell cycle seems a promising approach to therapy. Moreover, other targets have recently emerged including ependymal cells (Carlen et al., 2009) and glial precursors (Zhang et al., 2011). To our knowledge cell cycle of ependymal cells during neurodegeneration has not been assessed while some groups are now pioneering the study of gliogenesis.

## Gliogenic precursors

### Cell cycle in physiological conditions

Astrocytes and oligodendrocytes are the most abundant cell type of the adult brain with the latter gaining more interest for their role and potential use during brain recovery (Richardson et al., 2011; Tsai et al., 2012).

Oligodendrocyte progenitor cells (OPC) play pivotal roles in CNS development (Richardson et al., 2006) and adulthood where they represent the most abundant and homogeneously distributed cycling cell population of the CNS (Richardson et al., 2011). Oligodendrocytes during development are generated from different regions in consecutive waves but it is unknown whether each population has any specific role in brain function (Kessaris et al., 2006). Adult OPC represent 8 and 2% of the white and gray matter, respectively (Dawson, 2003; Rivers et al., 2008) with resident and migrating OPC in the SVZ and septum giving rise to mature myelinating oligodendrocytes in physiological and pathological conditions (Menn et al., 2006). Maturation of OPC involves changes in morphology and marker expression including Pdgfra, Ng2 for OPC and Olig2 and Sox10 for the whole lineage (Fumagalli et al., 2011). OPC are reactive to neurotransmitters (Bergles et al., 2000; Stevens et al., 2002) and display highly dynamic behavior with regard to migration, filopodia extension, proliferation, differentiation and reaction to injury (Hughes et al., 2013).

Starting at postnatal day 7 and during adulthood, approximately 50–80% of OPC in the whole brain were described as cycling based on BrdU incorporation (Rivers et al., 2008; Psachoulia et al., 2009; Simon et al., 2011). Another report based on EdU however calculated a growth fraction of about 99% (Young et al., 2013) but this thymidine analog has raised concern with regard to toxicity (Ponti et al., 2013). Cell cycle length of OPC differs among brain regions and is significantly longer than that of neurogenic precursors. In the white matter, in particular corpus callosum, OPC cell cycle linearly increases from 2 days at 1 week postnatal to a plateau of 150 days at 8 months (Rivers et al., 2008; Psachoulia et al., 2009; Young et al., 2013) with the spinal cord white matter yielding comparable results (Table 1) (Young et al., 2013). In the cortical gray matter cell cycle length was estimated to be about 37 days at 2 months (Simon et al., 2011; Young et al., 2013) with S/G2/M of 5 days (Simon et al., 2011). Cell cycle in the cortex also showed a decrease similar to the corpus callosum with the important difference that a plateau is not reached and cell cycle increases to up to 340 days at 18 months (Psachoulia et al., 2009; Young et al., 2013). This almost linear relationship between age and cell cycle implies a lengthening by about 16 h every day starting at birth (Young et al., 2013), that is, every cell cycle is two thirds longer than the previous one. Finally, cell cycle in the gray matter of the spinal cord is significantly shorter than in the cortex with OPC dividing every 8 or 27 days at 3 weeks or 2 months postnatal, respectively (Table 1) (Young et al., 2013). With regard to the proportion of OPC that divide to proliferate as opposed to generate mature oligodendrocytes, different studies led to different estimations while consistently reporting higher values for the white matter both in the brain and spinal cord with both declining during aging (Rivers et al., 2008; Psachoulia et al., 2009; Kang et al., 2010; Simon et al., 2011; Zhu et al., 2011; Young et al., 2013).

In conclusion, OPC exhibit a remarkably longer cell cycle than neurogenic precursors, which to our opinion reflects long periods of quiescence followed by re-entry in a cell cycle that is a fraction of the total inter-mitotic time. Moreover, the cell cycle of OPC differs between gray and white matter, which is possibly explained by region-specific differences as revealed by transplantation experiments (Vigano et al., 2013).

### Cell cycle in pathological conditions

Accumulating evidence indicates that OPC play key roles during brain injury (Nguyen et al., 2006; Huang et al., 2011; Zhang et al., 2013). Demyelination in multiple sclerosis leads to impaired saltatory signal conduction and loss of axon integrity (Huang et al., 2011). OPC react by migrating into the lesion and differentiate in mature myelinating oligodendrocytes and Schwann cells (Zawadzka et al., 2010). This reaction enhances recovery and is known to decrease with age making it a potential target for regenerative therapies (Nguyen et al., 2006; Zawadzka et al., 2010; Huang et al., 2011; Deshmukh et al., 2013).

Stab wound in the cortex increases proliferation in the whole brain with a five-fold higher response in the ipsilateral compared to contralateral hemisphere 3 days post injury (Simon et al., 2011). In particular, at 1 week 74% of OPC cycle suggesting that cells enter the cell cycle from quiescence and, concomitantly, shorten the G1-phase of their cell cycle (Simon et al., 2011).

Cerebral ischemia has a strong impact on oligodendrocytes since they lack the ability to proliferate and, once damaged, to myelinate axons (McTigue and Tripathi, 2008). After stroke resident and SVZ-derived OPC start to proliferate and migrate to the penumbra where they differentiate into mature oligodendrocytes that myelinate newly sprouted axons thus enhancing neuronal survival and short-term synaptic plasticity (Zhang and Chopp, 2009; Ueno et al., 2012; Zhang et al., 2013) and preclinical studies showed improved healing of stroke after pharmaceutically enhanced oligodendrogenesis (Zhang et al., 2013).

Most studies have focused on neuronal aspects of brain recovery and the role of other cell types awaits further investigation. Only recently studies started to focus on cell cycle parameters of OPC and other cell types such as astrocytes and pericytes playing critical roles in disease including glial scar formation and inflammation (Goritz et al., 2011; Lambertsen et al., 2012).

## Discussion

Decades of cell cycle measurements during development have been instrumental to understand and manipulate the contribution of neural precursors in the mammalian brain (Fujita, 1962; Schultze and Korr, 1981; Takahashi et al., 1995; Dehay and Kennedy, 2007; Salomoni and Calegari, 2010; Borrell and Calegari, in press). Studies during adulthood have just begun and parallelisms between the two contexts are hard to identify due to our limited understanding of adult lineages and difficulties in assessing cell cycle and quiescence. Notably, during development progenitors have longer cell cycles than stem cells (Borrell and Calegari, in press). Yet, differences of greater significance were found by comparing cells undergoing proliferative vs. differentiative division within these two populations (Calegari et al., 2005; Arai et al., 2011). Hence, analyses of, say, type 1/B vs. 2/C cells can only reveal part of the truth with identification of proliferative vs. differentiative precursors within each type being perhaps more important. Moreover, independently from physiological correlations between cell cycle and stemness, it is clear that artificial manipulations can still be effective in increasing stem cell expansion since these can override physiological processes as indicated by studies on NSC and OPC (Artegiani et al., 2011; Beukelaers et al., 2011a; Caillava et al., 2011; Nobs et al., 2013).

It is premature to know whether manipulation of neural precursors will ever allow practical and efficient means toward effective therapy, but the history of cell cycle measurements during development suggests that this may pave a promising road. To achieving this goal, technical limitations need first to be overcome including the identification of markers for the relevant cell types, establishing behavioral tests reflecting functional recovery rather than compensatory learning (Hicks et al., 2009) and animal models of disease faithfully recapitulating the human condition. As one example of the latter, models of stroke often involve the striatum whereas human ischemia mainly affects cortical areas while the few that involve the striatum cause mild deficits (Delavaran et al., 2013). Moreover, modeling disease is often done in young mice while most neurodegenerative diseases are relevant during aging, which has major effects on cell cycle and neurogenesis (Artegiani and Calegari, 2012). We envision that improvements in these aspects of biomedical research will have the greatest impact in the field and hope that this review will help readers to identify, hence overcome, some of our current limitations.

### Conflict of interest statement

The authors declare that the research was conducted in the absence of any commercial or financial relationships that could be construed as a potential conflict of interest.
